# Basal and Regulatory Promoter Studies of the AKR1C3 Gene in Relation to Prostate Cancer

**DOI:** 10.3389/fphar.2012.00151

**Published:** 2012-08-06

**Authors:** Jenny J. Schulze, Helena Karypidis, Lena Ekström

**Affiliations:** ^1^Department of Laboratory Medicine, Karolinska InstitutetStockholm, Sweden

**Keywords:** AKR1C3, 17BHSD5, prostate cancer, DHT, polymorphism, gene regulation

## Abstract

**Background:** Human 17β-hydroxysteroid dehydrogenase type 5 (17β-HSD5) formally known as aldo-keto reductase 1C3 (AKR1C3) play a major role in the formation and metabolism of androgens. The enzyme is highly expressed in the prostate gland and previous studies indicate that genetic variation in the AKR1C3 gene may influence the prostate volume and risk of prostate cancer. **Aim:** Here we aimed to further study the genetic regulation of AKR1C3 and its putative role in prostate cancer. **Experiments:** A previously identified promoter polymorphism (A>G, rs3763676) localized at −138 from the translational start site were studied in relation to prostate cancer in a Swedish population based case–control study including 176 patients diagnosed with prostate cancer and 161 controls. Moreover, we have studied the basal and androgen induced promoter activity of the AKR1C3 gene. Expression studies with AKR1C3 promoter reporter constructs were performed in HepG2 and DSL2 cells. **Results:** We found that carriers of the promoter A-allele had a borderline significant decreased risk of prostate cancer (OR = 0.59; 95% CI = 0.32–1.08). We also show that dihydrotestosterone (DHT) induced the promoter activity of the A-allele 2.2-fold (*p* = 0.048). Sp3 seem to play an important role in regulating the transcription activity of AKR1C3 and site-directed mutagenesis of a GC-box 78 base-pair upstream the ATG-site significantly inhibited the basal AKR1C3 promoter activity by 70%. **Conclusion:** These results further supports previous findings that the A>G promoter polymorphism may be functional and that AKR1C3 plays a critical role in prostate carcinogenesis. Our findings also show that the members of Sp family of transcription factors are important for the constitutive expression of AKR1C3 gene.

## Introduction

Human 17β-HSD type 5 belongs to the aldo-keto reductase (AKR) superfamily (Jez et al., 1997) and is formally known as AKR1C3. It is a promiscuous enzyme that participates in the biosynthesis and metabolism of a variety of substrates including androgens, estrogens (Penning et al., 2000), prostaglandins (Matsuura et al., 1998), and polycyclic aromatic hydrocarbons (PAH; Palackal et al., 2002). AKR1C3 is widely expressed in human tissues and is predominant in the prostate and mammary gland (Penning et al., 2000).

The 17-keto reductase activity of AKR1C3 reduces the weak androgen Δ^4^-androstenedione (4-dione) to testosterone. The combined 3α/3β activity of the enzyme (Steckelbroeck et al., 2004) partly inactivates 5α-dihydrotestosterone (DHT) to the weak androgens 3α-androstanediol (3α-Adiol) and 3β-androstanediol (3β-Adiol) and the 17β-hydroxysteroid oxidase activity of the enzyme oxidizes 3α-Adiol to androsterone instead of back to DHT (Penning et al., 2000).

The prostate gland is generally considered an important site of DHT formation and inactivation (Thigpen et al., 1993). DHT stimulates the proliferation of the prostate and AKR1C3 has been shown to be up-regulated in localized and advanced prostate adenocarcinoma (Nakamura et al., 2005; Fung et al., 2006; Stanbrough et al., 2006).

Previously an A>G polymorphism in the promoter region (rs3763676) at nucleotide position −138 from the translation start site was identified (Qin et al., 2006). The G-allele has been associated with lower promoter activity in human liver (HepG2), lung (A549), and prostate (LNCaP) cells (Jakobsson et al., 2007) and higher promoter activity in rat theca cells (Qin et al., 2006). The allele frequency of this SNP was shown to be significantly increased in patients with polycystic ovary syndrome (PCOS; Qin et al., 2006) and increased risk for bladder cancer (Figueroa et al., 2008) whereas its involvement in prostate cancer has not been studied.

Previous studies of the AKR1C3 promoter have shown that a 169-bp region (−104 to +65) is capable of directing transcriptional activity (Ciaccio et al., 1996). This 5′-flanking region contains a CTT-repeat element, two GC-boxes, and a reverse CCAAT-box. The GC- and CCAAT-boxes generally work as promoter signals in many eukaryotic cells. There is one study showing the Sp family of transcription factors play an important role in regulating constitutive expression of the AKR1C3 (Qin and Rosenfield, 2005).

The overall aim of this study is to increase the understanding on how AKR1C3 is genetically regulated and its putative role in prostate cancer. More specifically the aims of this study are to investigate (1) the influence of the CCAAT and the GC-elements on the basal expression of the human AKR1C3 gene in HepG2 cells, (2) investigate if DHT affect the transcriptional activity of AKR1C3 in relation to the promoter A>G polymorphism, and (3) the allele frequency of this promoter SNP in relation to risk for prostate cancer in a Swedish population.

## Materials and Methods

### Site-directed mutagenesis

Mutations in the CCAAT and GC-boxes were introduced using the pGLAKR1C3prom construct (Jakobsson et al., 2007) as template and the antisense primers (5′-gatggttaacatctggcatgtag-′3) and (5′-gaaacccctcccaacacccctg-′3) including the mutations together with a sense primer (5′-ggttgctatttgttctacaaa-′3). The PCRs were performed in 2 mM MgCl_2_, 0.15 mM dNTP, 0.3 μl Taq Polymerase, 20 pmol of each primer, 100 ng of template, and were carried out in 30 cycles, each involving denaturing at 94°, 45 s, annealing at 53°, 45 s, elongation at 72°, 1 min, followed by 7 min elongation. The PCR products were gel-purified (Qiagen) and used as a mega primer together with an antisense primer (5′-cattccctgtcacttgtctg-′3) in a second PCR. The product was subcloned into a TA-vector (Invitrogen) and cleaved with the restriction enzymes, *Xho*I and *Kpn*1 (New England BioLabs), gel-purified, and subcloned into similar digested pGL3 Basic vectors (Promega). All the mutations were verified by sequence analysis.

### Cell culture

Human hepatoma HepG2 cells were maintained at 37°C in 5% CO_2_ in MEM, 10% bovine serum, 2 mM glutamine, 1 mM sodium pyruvate, non-essential amino acids, 100 U penicillin, and 100 μg streptomycin/ml.

*Drosophila* DSL2 cells were maintained at room temperature in Schneider’s Medium, 10% bovine serum, 2 mM glutamine, 100 U penicillin, and 100 μg streptomycin/ml.

All cell culture media and their ingredients were obtained from GIBCO/BRL (Gaithersburg, MD, USA).

### Transfection assay

HepG2 cells were plated in 35 mm 6-well plates at 2 × 10^5^/well and incubated overnight. The cells were transfected using 15 μl Lipofectin (Life Technology), 2.5 μg pGLAKR1C3 Basic plasmid, and 2.5 μg β-gal control vector (Promega). Cells were incubated for 5 h at 37°C, the transfection solution was then replaced with fresh media. DSL2 cells were plated in 36 mm 6-well plates at 2 × 10^6^ cells/well at the day of transfection. Two micrograms of pGLBasicAKR1C3 plasmid, 2.5 μg β-gal control vector (Promega), 0.5–1.5 μg of Sp1, and/or Sp3 expression vectors (pPACSp1 and/or pPACSp3 kindly provided by Dr. Ahmed Zaid) and 10 μl Superfect (Qiagen) were used in each transfection reaction. Cells were incubated for 3 h at room temperature, the transfection solution were then replaced with fresh media. Cells were harvested 48 h after transfection and luciferase activity was determined using luciferase assay reagent (Promega). βgal activity was measured using *ortho*-nitrophenyl β-d-galactopyranoside (Sigma) as a substrate. All values were corrected for endogenous galactosidase activity. The luciferase values were divided by the βgal value in order to correct for transfection efficiency.

### Real-time PCR

Total RNA was extracted from DHT exposed HepG2 cells with Trizol reagent (Invitrogen) according to the manufacturers manual. 1 μg RNA was reverse transcribed using First-strand cDNA Synthesis Kit (Amersham) with a random hexamer oligonucleotide. Real-time PCR was performed using 1 μl cDNA, 10 pmol AKR1C3 specific primers (Bogason et al., 2011), SYBR Green (Applied Biosystems), and quantified in an ABI Prism 700. Dilutions of a plasmid containing AKR1C3 cDNA (19T7-AKR1C3Glu77; Jakobsson et al., 2007) was used to construct a standard curve. For normalization of cDNA input in each PCR, 18S mRNA was used as an endogenous control.

### Human study

In another part of the study a population based case–control study of 337 Caucasians living in the county of Örebro in Sweden were investigated. The cases were 176 patients, age 51–79 years, with prostate cancer, who were recruited consecutively between May 1994 and February 1996. In all, 81% agreed to participate. The controls were men, who were randomly selected every 3 months from the county population register and frequency matched for age. They were asked to participate by mail and 161 individuals agreed to participate, giving a response rate of 79%. Further description of the study population has been already published since this study population has been utilized in the evaluation of other polymorphisms involved in the androgen metabolism (Wadelius et al., 1999; Söderström et al., 2002). The Ethics Committee of the Regional Hospital of Örebro/Uppsala University, Sweden approved the study and all patients gave informed consent to participate. Genotyping of the A>G promoter polymorphism was performed as described previously (Jakobsson et al., 2007).

### Data analysis

Data of promoter activity and real-time PCR are expressed as mean ± SD Significance was assessed using two-tail Student’s *t*-test using GraphPad Prism software v4.03 (San Diego, CA, USA). Genotype associations were assessed with binary logistic regression using Minitab statistical software package (v 12.1, Minitab Inc., State College, PA, USA). ORs were used as an approximation of relative risk, using 95% confidence intervals.

## Results

### Functional analysis of human AKR1C3 basal promoter activity

**Figure 1:**
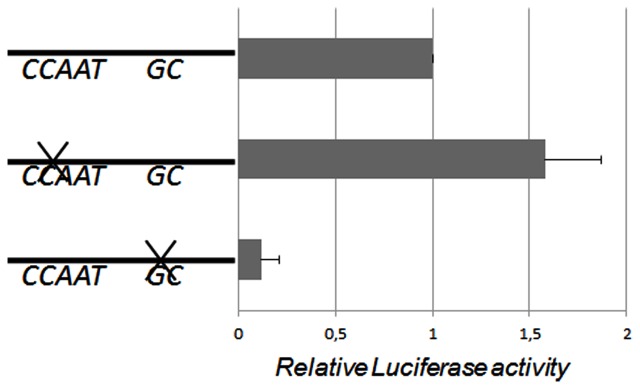
**Human AKR1C3 promoter fragment was subcloned into the pGL3 Basic vector and the CCAAT- and GC-elements were mutated and transiently transfected into HepG2 cells**. The bar graph represents mean ± SD from at least three experiments. The luciferase expression was normalized to the transfection efficiency by β-galactosidase expression. When the CCAAT-box was mutated there was a borderline significant 50% increase in the relative luciferase activity. When the GC-box was mutated the luciferase activity significantly decreased indicating that the GC-box is required for the basal transcription of the AKR1C3 gene in HepG2 cells.

Transfection assay with wildtype and mutant reporter constructs were performed in HepG2 cells to study the effect of the CCAAT- and GC-box mutations on luciferase activity. Mutation of the CCAAT-box did not inhibit the promoter activity in HepG2, instead a borderline (*p* = 0.06) significant 50% increase was observed (Figure 1).

Transfections with the reporter construct including a mutated GC-box indicate that the promoter activity decreased significant (70%, *p* < 0.05) in HepG2 cells (Figure 1). These results indicate that the GC-box is important for directing and activating the transcription of the AKR1C3 gene in HepG2 cells.

**Figure 2:**
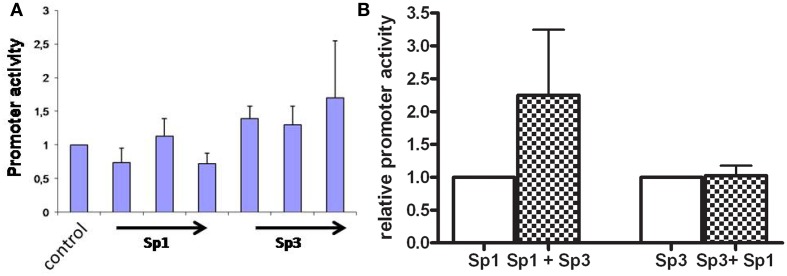
**(A)** Transcriptional activities of the AKR1C3 promoter construct transiently transfected into DSL2 cells. The DSL2 cells were cotransfected with Sp-proteins in increasing concentrations. The bar graph represents mean ± SD from at least three experiments. The luciferase expression was normalized to the transfection efficiency by β-galactosidase expression. Control background activity was increased twofold when Sp3 (0.5, 1, and 1.5 μg) were added to cells, whereas no significant affect was observed when increasing concentrations of Sp1 were added. **(B)** Human AKR1C3 promoter construct was transiently transfected into HepG2 cells cotransfected with Sp1/Sp3 proteins. The bar graph represents mean ± SD from at least three experiments. The luciferase expression was normalized to the transfection efficiency by β-galactosidase expression. When the Sp1 protein (1 μg) containing DSL cells were cotransfected with Sp3 (1.5 μg) the promoter activity increased 2.2-fold. When the Sp3 (1 μg) containing DSL cells were cotransfected with Sp1 (1.5 μg) no induction in promoter activity was observed.

Transfections with the wildtype promoter construct in DSL2 cells show that when increasing concentrations of Sp3 (0.5, 1.0, and 1.5 μg) were added to the cells, the promoter activity increased 1.5 times, whereas no activation of the basal activity of shown when increasing concentrations (0.5, 1.0, and 1.5 μg) of Sp1 were added (Figure 2A).

When Sp3 was added at 1 μg the addition of Sp1 (1 μg) did not further activate the promoter activity, whereas a Sp3 induced the promoter activity 2.2 (*p* < 0.05) times when added together with Sp1 (Figure 2B).

These results indicate that both the Sp1 and Sp3 proteins are involved in the transcription of AKR1C3 gene, although the Sp3 seem to be the most important protein.

### Steroid and promoter activity in relation to promoter polymorphism

**Figure 3:**
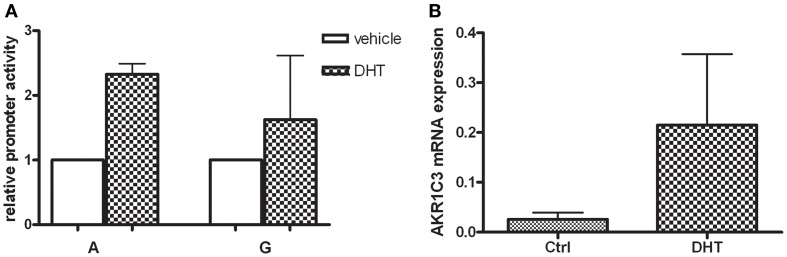
**(A)** The HepG2 cells were transfected with the wildtype AKR1C3 promoter (A) and the polymorphic (G) construct and exposed to 25 μDHT over night. The bar graph represents mean ± SD from at least three experiments. The luciferase expression was normalized to the transfection efficiency by β-galactosidase expression. After DHT treatment and promoter activity of the wildtype (A) significantly increased whereas no significant alteration was observed for the polymorphic (G) construct. **(B)** The mRNA expression of the human AKR1C3 gene in HepG2 cells was evaluated using real-time PCR. Significant increase in AKR1C3 mRNA levels was observed after incubation with 25 μM DHT over night. The bar graph represents mean ± SD from four experiments.

When the HepG2 cells were exposed to high concentration (25 μM) of DHT over night the promoter activity of the wildtype (A) construct increased 2.2-fold (*p* = 0.048), whereas for the polymorphic construct (G) there were no significant induction observed (Figure 3A). When HepG2 cells were exposed to DHT a significant sevenfold increase of the AKR1C3 mRNA levels (*p* = 0.038) was found (Figure 3B).

### Promoter polymorphism and prostate cancer risk

The distribution of the promoter polymorphism was in Hardy–Weinberg equilibrium. The allele frequency of the promoter polymorphism G in healthy participants were 39% (Table 1), as in agreement with previous studies in Caucasians (Jakobsson et al., 2007). Individuals displaying an A-allele (A/G and A/A) showed 0.59 times decreased risk for prostate cancer compared to individuals homozygous for the G-allele (Table 2). The results were quit not significant (*p* = 0.058).

**Table 1:** **Genotype and allele frequencies of the promoter polymorphism (A > G) in a Swedish population sample of prostate cancer and controls**.

	Cases % (*n*)	Controls % (*n*)
AA genotype	40 (71)	39 (63)
GG genotype	19 (33)	12 (19)
AG genotype	41 (72)	48 (77)

**Table 2:** **Prostate cancer risk estimates for AKR1C3 A/G promoter polymorphism among Swedish Caucasian men**.

Genotype	Odds ratio	95% CI
G/G	1.00	Ref.
A/G	0.54	(0.28–1.03)
A/A	0.65	(0.34–1.25)
A*	0.59	(0.32–1.08)

*All carriers of A compared to homozygous carriers of G.

## Discussion

To evaluate the possible role for AKR1C3 in prostate cancer, a promoter polymorphism (A>G, rs3763676) were genotyped in a population based case–control study. The result indicates that the promoter SNP in the AKR1C3 gene may modulate the prostate cancer risk and that there may be an advantage to have an A-allele. The results are in agreement with our *in vitro* finding that the promoter activity of the wildtype A variant is induced by DHT and with our previous finding that the G-variant displayed lower transcription activity in a prostate cancer cell line (Jakobsson et al., 2007). AKR1C3 is an important enzyme in the metabolism of DHT, particularly in the inactivation of DHT to the less potent androgen 3α-Adiol (Lin et al., 1997). It is possible that A-carriers may have an improved protection against high concentrations of DHT since this allele is correlated with higher transcriptional activity, and is induced by DHT. Contradictory to this hypothesis, i.e., that high expression of AKR1C3 is protective against androgen load, is a study by Stanbrough et al. (2006) who found a 5.2-fold increase of AKR1C3 mRNA level in androgen-independent prostate cancer bone metastasis.

Recent studies have shown that another AKR1C3 polymorphisms (c90 G/A (rs7741), known to be in linkage disequilibrium with the A>G promoter polymorphism (*R*^2^ = 1; International HapMap Consortium, 2005), is associated with prostate disease. The c90 A-allele has been associated with increased risk for prostate enlargement (Roberts et al., 2006), and increased risk of both familial and sporadic prostate cancer. (Cunningham et al., 2007). Additionally, another AKR1C3 SNP (rs4881400), not in linkage disequilibrium with the promoter polymorphism, has been associated with prostate cancer risk (Kwon et al., 2012). Thus it is likely that AKR1C3 play a role in the etiology of prostate related diseases. Whether the promoter polymorphism investigated in this study is associated with prostate cancer risk needs to be further investigated in larger prostate cancer case–control studies. The fact that the promoter SNP studied herein is included in common GWAS analysis such as Affymetrix SNP 6.0, will increase the chance to find such association.

In agreement with our results, one study found that the G-allele was significantly more frequent in women with PCOS. Like prostate cancer, PCOS is also considered to be an androgen dependent disease, and the authors speculated that the increased risk may be due to higher plasma testosterone levels in subjects homozygous for G-allele (Qin et al., 2006). However, a subsequent study were not able to find an association between the promoter A>G polymorphism and PCOS (Goodarzi et al., 2008).

Here we show that DHT increase the promoter activity of the AKR1C3 promoter including the wildtype (A) sequence, whereas the polymorphic G-variant was not up-regulated in HepG2 cells. Moreover, the AKR1C3 mRNA level was induced sevenfold after DHT exposure in HepG2 cells. This finding was not in agreement with a study performed in LNCaP cells, where DHT was shown to decrease the expression of AKR1C3 by 70% (Wang and Tuohimaa, 2007). This discrepancy of may be due to the different DHT concentrations used (10 nM versus 25 μM) in the studies. The former concentration is reflecting the physiological of DHT in the circulation, whereas the supra-physiologic concentration of 25 μM reflects the high levels of DHT produced locally in the prostate (Olsson et al., 2011). Moreover, the androgen response may differ between the cell-lines used. Further investigations are required to evaluate how androgens modulate expression and activity of AKR1C3 in different cell types, particularly in androgen sensitive cells.

In order to study the cis-acting elements involved in the AKR1C3 basal promoter activity we transfected the promoter wildtype and two mutated (CCAAT- and GC-elements) promoter construct in HepG2 cells. The result indicates that the inverted CCAAT-box is not involved in the basal transcription of AKR1C3. Instead binding to the CCAAT-box may have an inhibitory effect on the AKR1C3 expression since we observed an increase in promoter activity when this element was mutated. The GC-box on the other hand appears to be involved in the constitutive expression. The GC-boxes are GC rich sequences recognized by the Sp transcription factor family (Kingsley and Winoto, 1992). To further examine the trans-acting proteins involved in the AKR1C3 transcription, the wildtype promoter construct was transfected into DSL2 cells. DSL2 cells are used to specifically study the Sp family of transcription factors since these cells lack Sp-proteins. When constructs were added to the cells without the Sp1/Sp3 expression vectors, a low background activity was observed. The addition of Sp3 increased the expression, whereas the addition of Sp1 alone did not induce the promoter activity. Co-transfection with Sp1 and Sp3 proteins also indicate that Sp3 may have a more impact on the promoter activity compared to Sp1.

Previous studies of the AKR1C3 promoter have shown that a 169-bp region (−104 to +65) is capable of directing transcriptional activity (Ciaccio et al., 1996). This 5′-flanking region contains a CCT repeat element, 2 GC-boxes and a reverse CCAAT-box. Qin and Rosenfield (2005) showed that the binding of Sp1/Sp3 to the CCT repeat element was important for the constitutive and forskolin stimulated AKR1C3 promoter activity in H295R cells. The GC- and CCAAT-boxes generally work as promoter signals in many eukaryotic cells. The GC-boxes are GC rich sequences recognized by the Sp transcription factor family (Kingsley and Winoto, 1992). Our results together with Qins (Qin and Rosenfield, 2005) clearly show that the transcription of AKR1C3 gene is driven by Sp-proteins.

In conclusion, our results further support previous findings that AKR1C3 play an important role in the androgen metabolism and in the etiology of prostate cancer.

## Conflict of Interest Statement

The authors declare that the research was conducted in the absence of any commercial or financial relationships that could be construed as a potential conflict of interest.
